# Algae-Derived Bioactive Molecules for the Potential Treatment of SARS-CoV-2

**DOI:** 10.3390/molecules26082134

**Published:** 2021-04-08

**Authors:** Md. Asraful Alam, Roberto Parra-Saldivar, Muhammad Bilal, Chowdhury Alfi Afroze, Md. Nasir Ahmed, Hafiz M.N. Iqbal, Jingliang Xu

**Affiliations:** 1 School of Chemical Engineering, Zhengzhou University, Zhengzhou 450001, China; alam@zzu.edu.cn; 2 Tecnologico de Monterrey, School of Engineering and Sciences, Monterrey 64849, Mexico; r.parra@tec.mx; 3 School of Life Science and Food Engineering, Huaiyin Institute of Technology, Huaian 223003, China; bilaluaf@hyit.edu.cn; 4 Department of Biotechnology & Genetic Engineering, University of Development Alternative, Dhaka 1209, Bangladesh; chowdhuryalfiafrose@gmail.com; 5 Biotechnology & Natural Medicine Division, TechB Nutrigenomics, Dhaka 1209, Bangladesh; nasir.ahmedbd@hotmail.com

**Keywords:** antiviral agent, algae compounds, bioactive entities, COVID-19 treatment, immunomodulatory, therapeutic aspects, pharmacological uses

## Abstract

The recently emerged COVID-19 disease caused by severe acute respiratory syndrome coronavirus 2 (SARS-CoV-2) has adversely affected the whole world. As a significant public health threat, it has spread worldwide. Scientists and global health experts are collaborating to find and execute speedy diagnostics, robust and highly effective vaccines, and therapeutic techniques to tackle COVID-19. The ocean is an immense source of biologically active molecules and/or compounds with antiviral-associated biopharmaceutical and immunostimulatory attributes. Some specific algae-derived molecules can be used to produce antibodies and vaccines to treat the COVID-19 disease. Algae have successfully synthesized several metabolites as natural defense compounds that enable them to survive under extreme environments. Several algae-derived bioactive molecules and/or compounds can be used against many diseases, including microbial and viral infections. Moreover, some algae species can also improve immunity and suppress human viral activity. Therefore, they may be recommended for use as a preventive remedy against COVID-19. Considering the above critiques and unique attributes, herein, we aimed to systematically assess algae-derived, biologically active molecules that could be used against this disease by looking at their natural sources, mechanisms of action, and prior pharmacological uses. This review also serves as a starting point for this research area to accelerate the establishment of anti-SARS-CoV-2 bioproducts.

## 1. Introduction—Pandemic Problem and Opportunities

Algae biotechnology has been a great source of therapeutically useful molecules of supreme interests (e.g., proteins, peptides, amino acids, fatty acids, sterols, polysaccharides, oligosaccharides, phenolic compounds, photosynthetic pigments, vitamins, and minerals) [1,2]. Moreover, algae-derived bioactive molecules and/or compounds offer potential health benefits and diverse applications, such as antibacterial applications, antiviral applications, antifungal applications, anticancer applications, antidiabetic applications, anti-inflammatory applications, antioxidant applications, anti-obesity applications, neuroprotective applications, crop protection applications, and biofertilizer applications. This broad spectrum of the potential bioactivities of algae-derived bioactive molecules and/or compounds also offers great commercial possibilities in numerous industrial and biomedical sectors (such as pharmaceuticals, nutraceuticals, cosmeceuticals, etc.) [3,4]. Along with this, microalgae and their extracted multifunctional entities have been widely used as food in Asia, Africa, and South America for hundreds of years [5,6,7]. In Asia, marine microalgae have been used as a source for traditional food and folk medicine, such as in traditional Chinese and Indian medicine [8]. For instance, *Aphanizomenon flos-aquae* (AFA), a brackish and freshwater cyanobacterial species, is characterized by a drying quality and an ability to counteract an internal form of dampness in traditional Chinese medicine. It works on the kidney for diuretic or cleansing action. It also increases the production and release of natural killer (NK) cells against viruses and cancer cells. The consumption of AFA increases immune surveillance without directly stimulating the immune system [8,9]. The aqueous extracts of 10 marine algae (i.e., *Asparagopsis armata, Ceramium rubrum, Gelidium pulchellum, Gelidium spinulosum, Halopitys incurvus, Hypnea musciformis, Plocamium cartilagineum, Boergeseniella thuyoides, Pterosiphonia complanate*, and *Sphaerococcus coronopifolius* species of Rhodophyta from the coast of Morocco) were capable of inhibiting the replication of herpes simplex virus type 1 (HSV-1) in vitro at EC_50_ ranging from <2.5 µg mL^−1^ to 75.9 µg mL^−1^ without causing cytotoxic effects on the Vero cells [10].

Algae-derived bioactive compounds can be considered robust candidates for antiviral therapeutics. By taking into consideration the requisite therapeutic attributes, an array of microalgae strains has been screened to extract or isolate and purify new bioactive molecules [11,12]. To date, thousands of compounds derived from marine organisms have been screened and used for various therapeutic purposes. Among these, from the antiviral perspective, 21 have demonstrated antiviral activities against human enterovirus 71, human cytomegalovirus, human immunodeficiency virus type-1 (HIV-1), HSV, the influenza virus, the hepatitis B virus, murine norovirus, and respiratory syncytial virus (RSV), along with their modes of action **[13]**. Different crude extracts from Brazilian marine algae have also shown a high level of antiherpetic activity. In addition, the percentage of its antiviral activity against HSV-1 (86.1%) is higher than that against HSV-2 (55.5%) [14]. The aqueous extract of the red alga *Laurencia obtusa* has displayed an in vitro antiviral activity by inhibiting the replication of influenza B, A (H3N2), and A (H1N1) viruses [15]. Egyptian seaweed extracts of *Cystoseira myrica* and *Ulva lactuca* possess remarkable in vitro antiviral activities against a different virus-like hepatitis A virus, Coxsackie B4 virus, HSV-1, and HSV-2 [16]. The roles of several marine organisms and their metabolites in antiviral efficacy have been studied and reviewed [17,18,19,20,21,22]. However, limited literature is available on the COVID-19 related therapeutic usage of algae-derived, biologically active molecules. Thus, in aiming to present this unique and considerable pharmaceutical use of algae-derived biologically active molecules, an effort has been made herein to cover this gap in the literature concerning the antiviral activities and mechanisms of the actions of algae-derived bioactive molecules and/or compounds. Finally, an overview of the usage of algae-derived metabolites as biotherapeutics against SARS-CoV-2 causing COVID-19 is presented with suitable examples.

## 2. The Antiviral Activities of Algae-Derived Molecules

Marine-based molecules, such as carrageenan, agar, fucoidan, laminaran, and naviculan, have a high potential for use against viral infectivity. Furthermore, marine seaweeds and microalgae are high in amino acids and vitamins that can improve the immune system to fight against viral and bacterial diseases [23,24,25,26]. Marine algae contain sulfated polysaccharides that have been shown to inhibit the replication of enveloped viruses. Some other compounds (such as lectin, carrageenan, ulvans, and fucoidans from red, green, and brown algae) can act as biotherapeutic agents to prevent and cure COVID-19 [5]. The alga (e.g., *Cryptosiphonia woodii*) has a clinical efficiency against the herpes simplex virus [3]. A study on 39 marine red algae species has highlighted their potential usage as antiviral agents [27]. The method for using sulfated polysaccharides and carrageenan against retroviral infections, including HIV, has been reported in the literature. Carrageenan and iota-carrageenan are effective against enveloped and nonenveloped viruses and act as inhibitory agents to prevent the viruses from binding to host cells at the initial stages of infection to block HPV and HRV [28]. Along with this, carrageenan is the most commonly tested polysaccharide used against virus infectivity [29]. Carrageenan is generally recognized as safe (GRAS) and is approved as a food additive by experts from the US Food and Drug Administration [30]. Fucoidan, a potent natural sulfated polysaccharide extracted from two different macroalgae (*Dictyota bartayesiana* and *Turbinaria decurrens*), shows an inhibitory activity against HIV [31]. Table 1 represents a list of various marine algae-derived bioactive molecules and/or compounds (including their mechanisms of action) that exhibit potential antiviral properties against many human pathogenic viruses [32].

## 3. Algae-Derived Antiviral Polysaccharides and Mechanisms of Action

**Figure 1:**
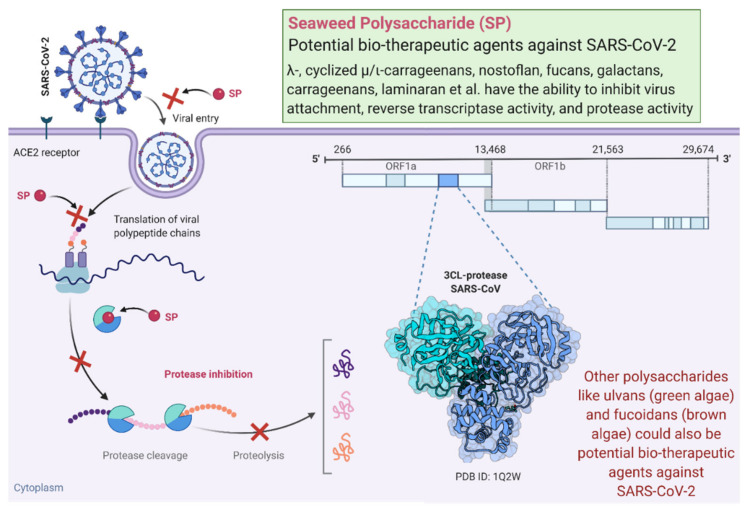
A schematic of seaweed polysaccharides (SP) used as notable biotherapeutic agents against SARS-CoV-2. The figure was created with the “BioRender.com” template and exported under the terms of premium subscription.

Marine organisms are rich sources of polysaccharides, and their antiviral activities were first reported over 50 years ago. Modified chitosan is a polysaccharide that significantly inhibits the human coronaviruses HCoV-229E, HCoV-OC43, HCoV-NL63, and HCoV-HKU1. Chitosan is an effective inhibitor of all low-pathogenic human coronaviruses [5,33]. Seaweed polysaccharides (SP) can inhibit the life cycle of a virus at different stages via direct inactivating virions before viral infection. Antiviral studies on polysaccharides such as carrageenan and chitosan have exhibited direct virucidal actions on some enveloped viruses to block viral infection [33]. The antiviral mechanisms of these polysaccharides underlie several processes, including the inhibition of viral absorption, the inhibition of virus internalization and uncoating, the inhibition of virus transcription and replication, and the improvement of host antiviral immune responses [33]. Figure 1 illustrates SP to be notable biotherapeutic agents against SARS-CoV-2. Dieckol is a phlorotannin that has been extracted from a brown alga *Ecklonia cava* and is reported as the most potent SARS-CoV 3CLpro trans-/cis-cleavage inhibitory activity in a dose-dependent and competitive manner with no toxicity [34]. Sulfated polysaccharides (such as fucoidan and sulfated rhamnan) can interfere or inhibit the expression and activation of the epidermal growth factor receptor pathway, which may suppress coronavirus [35]. Sulfated polysaccharides have unique antiviral mechanisms that function as direct inhibitors of retroviruses (including HIV) and are considered a “new generation antiretroviral drug” [31].

## 4. Potential of Algae-Derived Antiviral Metabolites against SARS-CoV-2

The marine alga *Halimeda tuna-*derived halitunal, a novel diterpene aldehyde, shows antiviral effects against murine coronavirus A59 in vitro [36]. Red alga-derived Griffithsin has antiviral effects that bind to oligosaccharides on the surfaces of various viral spike glycoproteins, including SARS-CoV [37] and MERS-CoV [38]. Griffithsin inhibits a broad range of CoVs, including HCoV-229E, HCoV-OC43, and HCoV-NL63 in vitro and SARS-CoV-infected mice [39]. Therefore, Griffithsin may be effective against SARS-CoV-2 because it can inhibit virus entry, reverse transcriptase activity, integrase activity, and protease activity [40]. Other sulfated polysaccharides, such as ulvans (derived from green algae) and fucoidans (derived from brown algae), are also considered to be potential biotherapeutic agents useful against SARS-CoV-2 [5]. An *in silico* study on the antiviral potential of *Arthrospira*-derived metabolites against SARS-CoV-2 was performed with three identified molecules (i.e., phycocyanobilin, phycoerythrobilin, and folic acid) that show the binding capability required to compete with SARS-CoV-2 [41]. Another in silico experiment was performed on eight algae-derived compounds obtained from three different red macroalgae (namely, *Laurencia papillosa, Gracilaria corticata*, and *Grateloupia filicina*) to screen therapeutic SARS-CoV targets, and these compounds can also be used for further in vitro and in vivo studies to search for antiviral agents that inhibit SARS-CoV-2 **[42]**.

Spirulina, a type of nutritious blue–green algae rich in phenolic acids, essential fatty acids, sulfated polysaccharides, and vitamin B12, is a commercially available food supplement. It shows effective antiviral activities against pseudo-type coronaviruses by binding to the S1 domain of 36 spikes and blocking the interaction of spikes with its receptor [43]. Sulfated polysaccharides derived from red algae *Porphyridium* sp. are promising antiviral agents that can be used as a coating material on sanitary items for COVID-19 prevention [44]. Another study [45] has suggested the use of the microalga *Haematococcus pluvialis*-derived natural astaxanthin (nASX) as adjunctive in combination with primary antiviral drugs that will largely benefit patients with COVID-19 by improving their health and reducing their recovery time. Astaxanthin is considered GRAS and is approved by the United States Food and Drug Administration for human consumption [45]. A review study has also been performed to provide a novel idea for the development of anti-SARS-CoV-2 drugs and vaccines by using a combination of polysaccharides and nanotechnology [35]. Table 2 summarizes marine algae-derived metabolites with potential against 2019-nCoV.

## 5. Algae-Derived Metabolites for Microbiota-Based Therapy and Immunomodulatory Activity against SARS-CoV-2

The catastrophic effects of SARS-CoV-2 include gastrointestinal (GI) symptoms. Approximately 20% of patients with SARS-CoV-2 exhibit GI problems, as reported by a Hong Kong cohort study [46]. Another study analyzed 95 patients with this virus [47]. Effenberger et al. [48] found that fecal calprotein is higher in SARS-CoV-2-infected patients, and 61% of them suffered from GI issues such as diarrhea and nausea. A recent pilot study involving shotgun metagenomic sequencing has been performed to investigate the microbiome composition of stool samples from 15 hospitalized patients with COVID-19 and compared it with those of healthy noninfected individuals; this study demonstrated the association between poor gut health and the severity of SARS-CoV-2 infectivity [49]. This study also found that patients with COVID-19 have higher levels of the harmful microbes *Actinomyces viscosus, Bacteroides nordii*, and *Clostridium hathewayi* and lower levels of the friendly microbes *Faecalibacterium prausnitzii, Lachnospiraceae bacterium, Eubacterium rectale, Ruminococcus obeum,* and *Dorea formicigenerans (*some of which have immunomodulatory and anti-inflammatory properties) [49].

**Figure 2:**
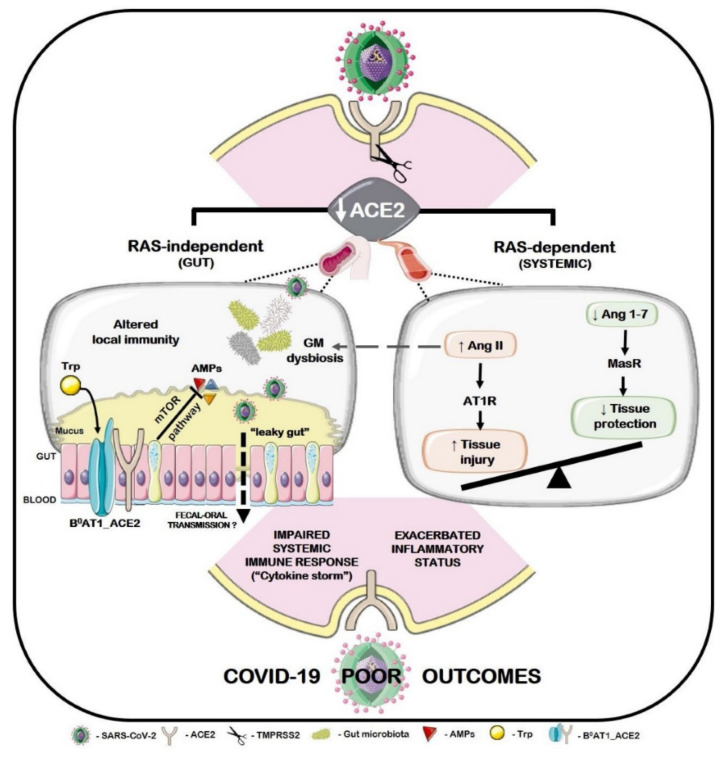
The putative association between ACE2 shedding after SARS-CoV-2 infection. Reprinted from Viana et al. [56] with permission from Elsevier. Copyright 2021, Elsevier. License Number: 4993920853888.

**Figure 3:**
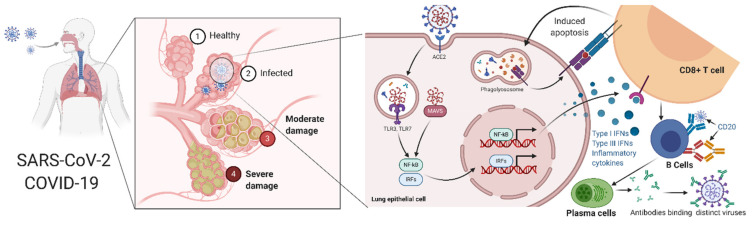
A schematic of the COVID-19 viral immune response and the targets of common dermatologic immunomodulators. The SARS-CoV-2 virus attacks host cells by binding to their receptors present in the cell membrane. Upon the first infection, lung epithelial cells become the primary target, where the receptor-binding domain of the virus spikes binds to ACE2 receptors. SARS-CoV-2 infects the human lung epithelium via the receptor ACE2. Viral RNA activates endosomal and cytoplasmic sensors (TLR3/7 and MAVS, respectively). These receptors activate interferon regulatory factors (IRFs) and NFkB to induce inflammatory cytokines, including interferons (IFN). CD8 T cells induce apoptosis after the recognition of antigens on infected cells. Conversely, activated B cells differentiate into plasma cells that produce antibodies important for neutralizing viruses. This figure was created with the “BioRender.com” template and exported under the terms of premium subscription.

Nevertheless, a healthy gut microbiome is crucial for its supportive role in antiviral immunity [50], and improving gut flora by maintaining a nutritional diet is essential for strengthening the gut microbiota to attenuate the effect of novel SARS-CoV-2 [51]. Gut microbiome dysbiosis, which is known for the resultant alterations of gut microbiota, has also been shown as related to several diseases and disorders, such as depression [52], obesity [53], IBS [54], and type-2 diabetes **[55]**. Gut microbiota dysbiosis may be associated with abnormal angiotensin-converting enzyme 2 (ACE2) functions that play a critical role in patients with COVID-19 and pre-existing age-related comorbidities [56]. The putative association between ACE2 shedding after SARS-CoV-2 infection is shown in Figure 2 [56]. By contrast, healthy gut microbiota-derived metabolites improve antiviral immunity by stimulating interferon production, decreasing immunopathology, and increasing natural killer (NK) cytotoxicity [57]. Figure 3 schematically shows the viral immune responses to COVID-19 and targets common dermatologic immunomodulators.

Marine algae-derived bioactive compounds, such as alginates, fucoidans, luminaries, polyphenols, carrageenans, carotenoids, fatty acids, and phlorotannins, offer benefits to human gut microbiota that maintain the host health by regulating the development and function of metabolism, the epithelial barrier integrity, and the immune system [8,58] as prebiotics or nutritional and functional food [59]. A diet containing a balance of vitamins and minerals as immune nutrients can also be a considerable strategy to fight against COVID-19 [60], and algal seaweed-based foods are rich in vitamins (Table 3), useful for potential supplementation in consideration of hypervitaminosis risk factors. Moreover, the immunological activity of phytosterols, carotenoids, vitamins, and fatty acids extracted from different microalgae species has been demonstrated extensively [61]. Consuming the green alga *Chlamydomonas reinhardtii* can improve the human gastrointestinal issues associated with irritable bowel syndrome (IBS) (such as diarrhea, gas, and bloating) and has shown no signs of causing dysbiosis or an adverse effect on microbiota composition [62]. *Spirulina* modulates several immune functions in the gut and upregulates the expression of the toll-like receptors 2 and 4 (TLR2 and TLR4) in the ileum of aged mice [63]. *Spirulina maxima*-derived modified pectin modulates gut microbiota and triggers immune responses in mice [64]. The supplementation of algae-derived β-glucan alleviates diarrhea and enhances gut health in *E. coli*-infected weaned pigs [65]. Sulfated polysaccharides from the brown seaweed *Ascophyllum nodosum* have uses as a functional food to modulate the composition of gut microbiota and increase the abundance of beneficial *Bacteroidetes* and *Firmicutes* [66]. Algae-derived undigested polysaccharides, alginate, agarose, and carrageenan have been shown to benefit the structure of human gut microbiota and gut health [67]. Digested extracts from the seaweeds *Sargassum muticum* and *Osmundea* have been used in novel functional foods and can possibly benefit the human gut microbiome [68]. Thus, improving the gut microbiome and its metabolites and implementing a personalized diet can be a considerable strategy in preventing and treating the novel coronavirus causing COVID-19 [69].

## 6. Algae-Derived Glycan Therapeutics against SARS-CoV-2

Glycoproteins are oligosaccharide glycan chains containing glycoconjugates of proteins with a sugar attached to them, and are covalently attached to amino acid chains generated by glycosylation. Glycoproteins have an extensive range of biological activities, including antiviral properties [76]. Glycotherapy has the potential to be a pioneering future biotherapeutic breakthrough. Glycoprotein-based antiviral therapy is an emerging novel research paradigm [77] precisely because an initial attachment between an enveloped virus and a host cell occurs via the interaction between the spike (S) glycoproteins and the glycans of the cell surface glycoproteins [78].

The glycosylation of the viral envelope proteins is essential for infectivity and can affect immune recognition, and some clinical applications are developed through this glycosylation process [79]. The glycosylation of epitope masking has been observed in coronavirus spike proteins. These proteins are large glycoproteins with 23–38 N-linked glycan sites per protomer [80]. Another study [81] has been performed to identify the location of 22 glycosylation sites, where glycans are attached to the SARS-CoV-2 spike and form sugar at each site. Wrapp et al. [82] reported that the S-glycoprotein of SARS-CoV-2 contains 66 glycosylation sites. TMPRSS2 (a human serine protease) is responsible for priming the S-glycoprotein of SARS-CoV-2, and the human angiotensin-converting enzyme 2 (hACE2) is engaged as a receptor for the entry of SARS-CoV-2 [83]. Lectins (hemaglutinins) are a diverse group of unique carbohydrate-binding glycoproteins that bind reversibly to monosaccharides and oligosaccharides with a high specificity [84], and various red algae are reported to possess lectins that have high-mannose N-glycans [85]. Lectins inhibit coronavirus infectivity [86,87] and are specific for the glycans present within the SARS-CoV spike glycoprotein, which has N-glycosylation. Some particular plant lectins are reported to be promising against SARS-CoV-2 [88]. The red algae-derived high-mannose-binding lectin of 121 amino acids (namely, Griffithsin (GRFT)) is reported to inhibit SARS-CoV entry by specifically binding to the spike glycoprotein [37,39]. GRFT has been considered as a superior broad-spectrum antiviral therapeutic that demonstrates a potent antiviral action with the least amount of host toxicity against various enveloped viruses [40]. Some reported marine algae-derived antiviral lectins are summarized in Table 4.

## 7. Algae-Derived Antioxidants against SARS-CoV-2

**Figure 4:**
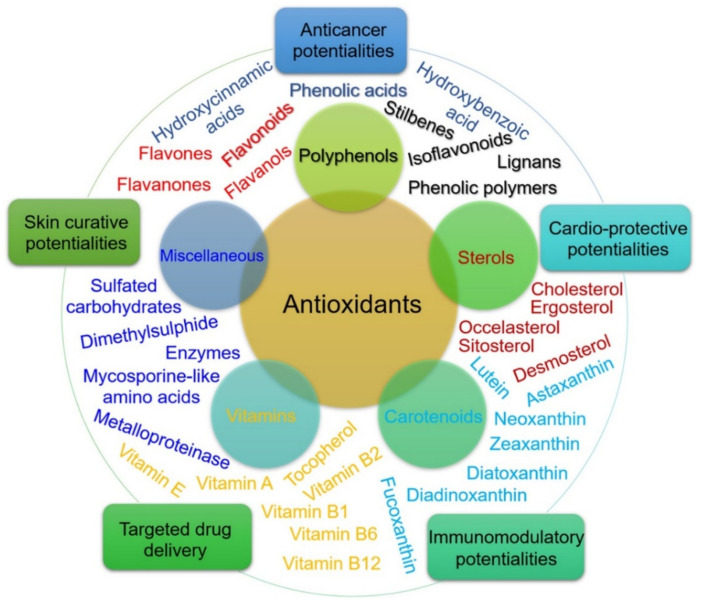
An array of algae-based antioxidants. The inner circle shows the diversity of biologically active antioxidants produced in a marine environment. The outer circle represents their novel therapeutic and biomedical potentialities. Reprinted from Bilal and Iqbal [101] with permission from Elsevier. Copyright 2021 Elsevier B.V. License Number: 4982940408845.

Antioxidants are compounds that can prevent oxidative stress (OS), which promotes viral infections, and they, along with antiviral agents, have a role in treating viral diseases (Figure 4) [101]. The oxidative stress that has been shown in HIV and influenza infections in humans and reactive oxygen species (ROS) produced by free radical actions is believed to activate viral replication [102]. OS is often defined as a disrupted balance of ROS production, which plays a key role in the normal functionality of the immune system [103]. Therefore, the T cell response is ultimately induced, and an overall immunological defense is enhanced [104].

Natural products derived from marine and freshwater organisms have been reported to exhibit antioxidant activities against some respiratory viruses [105]. These viruses enhance ROS production and effect cellular defense systems against ROS, which are implicated in inflammation, lung and tissue damage, and epithelial dysfunction, and the vital role of ROS exists in SARS-CoV [106]. In a review [107], oxidative stress (OS) is the key player in SARS-CoV infection, and high ROS levels are related to oxidative cell damage, which may be a result of increased inflammation at the location of viral infection that disrupts antioxidant mechanisms, leading to an unbalanced oxidative–antioxidant status, and subsequently triggering OS-causing cellular damage. SARS-CoV 3CLpro significantly increases in ROS production in HL-CZ cells by activating the NF-kB-dependent reporter gene, and the ROS-activated NF-kB signal transduction pathway can be considered a key player in SARS-CoV infectivity [107]. Other studies have suggested that OS is a significant factor for increasing the severity of SARS-CoV-2 infection because it triggers an inflammatory reaction and have recommended the use of antioxidants in therapeutic strategies against COVID-19 [108,109]. Natural antioxidants from marine algae derivatives are a rich source of ROS scavenger compounds belonging to vitamins, active peptides, carotenoids, sulfated polysaccharides chito-oligosaccharides and their representative derivatives, sterols, phlorotannins, phenolics, and flavones with unique structural and functional attributes [110]. Table 5 represents a list of antioxidant compounds derived from marine algae, and Table 6 shows a list of biologically active compounds with antioxidant activities in algae extracts [111,112,113,114,115,116].

## 8. Conclusions

The pandemic caused by SARS-CoV-2 has resulted in severe devastation worldwide. Scientists and global health experts have collaborated to find and execute speedy diagnostics, robust and highly effective vaccine development and deployment, and novel therapeutic techniques to tackle COVID-19 disease. A large source of natural marine compounds with biopharmaceutical activities associated with antiviral and immunostimulatory properties and some specific algae-derived antiviral metabolites is the ocean. Marine-derived biologically active constituents can also be considered a unique source of antibodies and vaccines against SARS-coV-2. Marine algae and seaweeds are the ancestors of all plants. Algae-based biotechnology has provided many opportunities and highlighted the potentiality of immense innovations with low-cost technologies for developing and revolutionizing bio-based industries. Algae and other marine organisms produce varied biometabolites that help them acclimatize and survive under harsh conditions. This review reveals that these specialized natural metabolites can be a great source of antiviral agents. These bioactive agents have been evaluated and exploited as microbiota-based therapeutic agents, immunomodulators, glycan therapeutic agents, and antioxidants for their biotherapeutics in preventing and treating SARS-CoV-2. Indeed, the above discussed literature evidences prove that marine algae-derived metabolites have a high efficiency against SARS-CoV-2. In this context, further research and clinical studies should be conducted to develop the most effective natural biotherapeutics from marine algae-based sources.

## Figures and Tables

**Table 1:** The antiviral activities and mechanisms of actions of algae-derived bioactive molecules and/or compounds.

Algae Spp.	Antiviral Bioactive Compounds	Structures	Active against Viruses	Mechanism of Actions
*Gigartina skottsbergii*	Carrageenan	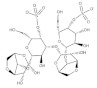	Influenza virus, DENV, HSV-1, HSV-2, HPV, HRV, HIV	Inhibition of the binding or the internalization of viruses into host cells (Stage I, II, III) *
*Callophyllis variegata*, *Agardhiella tenera*, *Schizymenia binderi*, *Cryptonemia crenulata*	Galactan	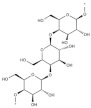	HSV-1, HSV-2, HIV-1, HIV-2, DENV, HAV	Blockage of virus adhesion and replication into host cells (Stage I, III)
*Laminaria hyperborea*, *Laminaria digitata*, *Laminaria japonica*,	Alginate	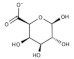	HIV IAV, HBV	Inhibition of the inverse transcriptase in the RNA virus (Stage III)
*Adenocytis utricularis*, *Undaria pinnatifida*, *Stoechospermum marginatum*, *Cystoseira indica*, *Cladosiphon okamuranus*, *Fucus vesiculosus*	Fucan	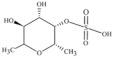	HSV-1, HSV-2, HCMV, VSV, Sindbis virus, HIV-1	Inhibition of cell adhesion (Stage I); blockage of reverse transcriptase (Stage III)
*Fucus vesiculosus*, *Saccharina longicruris*, *Ascophyllum nodosum*	Laminaran	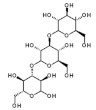	HIV	Blockage of reverse transcriptase (Stage III)
*Chlorella vulgaris,Cochlodinium polykrikoides*, *Porphyridium* sp.	Sulfated polysaccharides (e.g., Agar)	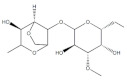	Influenza A and B viruses, RSV-A, RSV-B, parainfluenza-2	Inhibition of the cytopathic effect (Stage II); inhibition of PMN migration toward chemoattractant molecules; partial blocking of the adhesion to endothelial cells
*Dunaliella primolecta*	α- and β-Pheophorbide	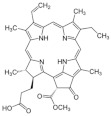	HSV-1	Inhibition of adsorption and invasion (Stage I, II)

* Stage I = virus adhesion, adsorption, entry, and invasion of cells; stage II = the cell is forced to replicate multiple copies of the virus genome; stage III = maturity and release of the virus particles (virions).

**Table 2:** Marine algae metabolites against novel human coronavirus (2019-nCoV).

Name of Algae-Derived Metabolites	Source Organisms
Griffithsin	Red algae, *Griffithsia*
Ulvans	Green algae, *Ulva* sp.
Fucoidans	Brown algae
Phycocyanobilin, phycoerythrobilin, and folic acid	*Arthrospira*
Sulfated polysaccharides	Red algae *Porphyridium* sp.
Astaxanthin (nASX)	*Haematococcus pluvialis*
Spirulina	*Arthrospira platensis*
Halitunal	*Halimeda tuna*
Dieckol	*Ecklonia cava*
n-Hexadecanoic acid, hexadecenoic acid, methyl ester, n-decanoic acid, and 9-dodecenoic acid	*Laurencia papillosa, Gracilaria corticata*, and *Grateloupia filicina*

**Table 3:** Algae-derived vitamins as sources of immune nutrition.

Vitamins	Source	Reference
Vitamin C	*Porphyra umbilicalis, Himanthalia elongata, Laminaria spp., Gracilaria changii, Palmaria palmata Eisenia arborea J.E. Areschoug*	[70,71,72]
Vitamin B1	*Tetraselmis suecica, Isochrysis galbana, Dunaliella tertiolecta, Chlorella stigmatophora, Chondrus ocellatus*	[73,74]
Vitamin B2	*Tetraselmis suecica, Isochrysis galbana, Dunaliella tertiolecta, Chlorella stigmatophora*	[73]
Vitamin B12	*Tetraselmis suecica, Isochrysis galbana, Dunaliella tertiolecta, Chlorella stigmatophora*	[73]
Vitamin E	*Laminaria spp., Porphyra umbilicalis, Himanthalia elongata, Palmaria palmata, Eisenia arborea J.E. Areschoug, Tetraselmis suecica, Isochrysis galbana, Dunaliella tertiolecta, Chlorella stigmatophora*	[70,71,73]
α-tocopherol (vitamin E)	*Macrocystis pyrifera, Gracilaria chilensis, Codium fragile*	[75]
β-carotene	*Gracilaria changgi, Laminaria spp., Porphyra umbilicalis, Gracilaria chilensis, Codium fragile, Tetraselmis suecica, Isochrysis galbana, Dunaliella tertiolecta, Chlorella stigmatophora*	[70,72,73,75]

**Table 4:** List of some reported marine algae-derived antiviral lectins.

Lectin Designated	Marine Algae Source	Virus	References
GRFT	*Griffithsia* Sp.	SARS-CoV, HIV, HCV	[37,89,90]
Microvirin	*Microcystis aeruginosa*	HIV-1	[91]
Cyanovirin	*Nostoc ellipsosporum*	HIV	[92]
AML, BSL, HML, MEL, Sfl	*Amansia multifida*, *Bryothamnion seaforthii*, *Hypnea musciformis*, *Meristiella echinocarpa* and *Solieria filiformis*	HIV and influenza	[93]
ESA-2	*Eucheuma serra*i	Influenza	[94]
KAA-2	*Kappaphycus alvarezii*	Influenza	[95]
BCA	*Boodlea coacta*	Influenza, HIV	[96]
HRL40	*Halimeda renschii*	Influenza	[97]
MVL	*Microcystis viridis*	HIV-1	[98]
Scytovirin	*Scytonema varium*	HCV, HIV, Ebola	[99,100]

**Table 5:** The bioactive compounds extracted from marine algae with significant antioxidant potentialities.

Antioxidant Compound	Microalgae Source	References
Chlorophyll-a derivatives, pheophorbidea	*Enteromorpha prolifera*	
Fucoxanthin	*Undaria pinnatifida, Turbinaria ornata*	[111,112]
Phycoerythrobilin	*Porphyra* sp.	
β-carotene	*Dunaliella salina, Chondrus crispus, Mastocarpus stellatus*	
Astaxanthin, canthaxanthin, lutein	*Haematococcuspluvialis*	
Lutein, violaxanthin	*Chlorella pyrenoidosa*	[113,114]
Canthaxanthin, astaxanthin	*Chlorella vulgaris*	
Phenolic and flavonoids	*Nannochloropsis oculata*, *Gracilaria gracilis*	[115]
Phycoerythrin, phycocyanin	Red algae	
Terpenoids	*Cystoseira* sp.	
Sulfated polysaccharides (Fucoidan, alginic acid, laminaran, sulfated galactans, galactans, sulfated glycosaminoglycan, porphyran	*Turbinaria conoides, Laminaria japonica, red algae, Sargassum wightii, Porphyra* sp.	
Stypodiol, taondiol, isoepitaondiol	*Taonia atomaria*	[116]

**Table 6:** Algae extract-derived compounds with antioxidant activities.

Vitamins	Ascorbate (vitamin C)	
	Tocopherol (vitamin E)	α-, γ-, δ- tocopherol
Carotenoids	α-carotene and β-carotene	
	Fucoxanthin and astaxanthin	
Polyphenols	Phlorotannin - Brown algae polyphenol	Fucol Phlorethol Fucophlorethol Fuhalol Isofuhalol Eckol
	Catechin	Catechin (3-hydroxyflavone)/catechin gallate Epicatechin/epicatechin gallate Epigallocatechin/epigallocatechin gallate
	Phenolic acid	
	Flavonoids	Anthocyanins Flavonols Flavanols Flavanones Flavones Isoflavones
	Tannins	
	Lignans	
Mycosporine-like amino acids	Mycosporine–glycine	
